# Challenges of managing people with multimorbidity in today’s healthcare systems

**DOI:** 10.1186/s12875-015-0344-4

**Published:** 2015-10-14

**Authors:** Keith Moffat, Stewart W. Mercer

**Affiliations:** Institute of Health and Wellbeing, College of Medical, Veterinary and Life Sciences, University of Glasgow, Glasgow, G12 9LX UK

## Abstract

Multimorbidity is a growing issue and poses a major challenge to health care systems around the world. Multimorbidity is related to ageing but many studies have now shown that it is also socially patterned, being more common and occurring at an earlier age in areas of high socioeconomic deprivation. There is lack of research on patients with multimorbidity, and thus guidelines are based on single-conditions. Polypharmacy is common in multimorbidity, increasing drug-disease and drug-drug interactions. Multimorbid patients need holistic care, but secondary care services are highly specialised and thus are often duplicative and fragmented and thus increase treatment burden in multimorbid patients. The cost of care is high in multimorbidity, due to high rates of primary and secondary care consultations and unplanned hospital admissions. The combination of mental and physical conditions increases complexity of care, and costs. Mental-physical multimorbidity is especially common in deprived areas.

General practitioners and primary care teams have a key role in managing patients with multimorbidity, using a patient-centred generalist approach. Consultation length and continuity of care may need to be substantially enhanced in order to enable such patients. This will require a radical change in how health care systems are organised and funded in order to effectively meet the challenges of multimorbidity.

## Background

Multimorbidity, commonly defined as the co-existence of two or more chronic conditions within an individual [1], is now the norm in ageing populations around the world [2]. Furthermore, it is socially patterned, occurring more often and at an earlier age in patients of lower socioeconomic status (SES) [2, 3]. Thus multimorbidity should not be considered exclusively as an issue of older age, and affects many people of working age [3, 4]. There are multiple challenges in managing patients with multimorbidity, some of which are discussed below.

### Evidence base and guidelines

Research and guidelines on the management of long term conditions has routinely focused on single diseases [2, 5]. Patients with multimorbidity are usually excluded from randomised controlled trials [3, 6]. This has led to individual disease management rather than a more holistic approach [5]. A recent systematic review of interventions for patients with multimorbidity found only ten studies worldwide [7]. There was a particular dearth of studies in high deprivation settings, or that focused on patients with low SES [7].

There is a thus an urgent need for more interventions to be tested in pragmatic trials in multimorbid populations, especially in relation to health inequalities, and for these to inform future guidelines. A NICE guideline on the clinical assessment and management of multimorbidity is expected to be published next year [8]. Although this will provide welcome guidance to this complex area of practice, the advice is likely to be generic rather than specific, given the paucity of research to date.

### Polypharmacy

Drug therapy in multimorbidity is a common area of difficulty for both patients and physicians [5, 9, 10]. Polypharmacy is common in multimorbidity because guidelines are single-disease focused and advise when to start new drugs but seldom when to stop them. The more LTCs (long term conditions) a patient has the more medications they are likely to be prescribed [4, 11, 12]. Polypharmacy commonly leads to drug-disease interaction and drug-drug interactions [10, 13].

### Specialism

Healthcare systems are largely based around a single-disease paradigm and thus specialist care of the multimorbid patients is often fragmented and duplicative with an increasing trend toward super-specialism [1, 3, 14]. This can create multiple problems and barriers to holistic patient centred care. The pivotal role of generalism in the management of patients with multimorbidity is becoming increasingly evident. Although in some systems this function can be provided by a general physician or internist, in countries with a well-developed primary care system, such as in the UK, much of this role depends on general practitioners (GPs) [15]. Expert generalist care is not just medical care for several conditions, but crucially combines the biotechnical with the biographical, in what has been termed interpretive medicine [15] in which a patient-centred approach is tailored to each patients circumstances and choices.

### Treatment burden

Treatment burden describes the demand which patients and their caregivers are placed under by the healthcare system [16]. This is common in patients with multimorbidity as they manage an increasingly chaotic medical lifestyle. They must negotiate their way through multiple fragmented appointments, investigations and medication regimes. As well as being disruptive for the patient this can also affect adherence [17]. The solution is “minimally disruptive medicine” which aims to reduce the workload of managing illness by better co-ordinating care and emphasising patient choice [17].

### Resources

Managing multimorbidity is hard work for patients [18, 19], and for practitioners [9, 19], especially when compounded with socioeconomic deprivation. Managing patients with multimorbidity is also financially costly. The more long term conditions a patient has then the greater their use, and thus cost, of health care. This includes primary care, secondary care outpatient visits, and hospital admissions [20]. This is also true of potentially avoidable acute admissions, which are increased by multimorbidity, deprivation and mental health problems [21]. There is a growing recognition that with increasing levels of multimorbidity the sustainability of current healthcare systems around the world is under threat [22].

### Mental-physical multimorbidity

Mental health problems such as depression are known to be common in patients with multimorbidity and the prevalence of mental health problems increases in a linear way with increasing numbers of physical conditions within individuals [3, 23]. This has several negative consequences, including the ability of the patient to manage their conditions. Mental-physical multimorbidity is 2–3 times more common in patients living in deprived areas compare to those in affluent areas, and thus presents the GP with increased complexity [1, 24]. Recent work suggests that a collaborative care model may help patients with mental-physical multimorbidity in primary care [25].

### Primary care systems

It is commonplace for general practices in the UK to offer 10 min appointments to patients regardless of the complexity of their health problem. This is unlikely to be long enough to comprehensively deal with the complex issues which arise in multimorbidity. In addition, in areas of high deprivation the inverse care law continues to exist, leading to shorter consultations, less patient enablement and higher GP stress [26, 27]. Giving longer consultations to patients with complex needs in deprived areas can increase patient enablement and reduce GP stress [28].

Continuity of care is an important part of the management of patients with complex health conditions [29]. Patients value seeing the same doctor [30], however this has become more difficult with the primary care reforms in the UK [31].

## Conclusion

Patients with multimorbidity have complex healthcare needs. There are many challenges faced in the management of multimorbidity, requiring a holistic approach by a generalist in order to balance the often competing priorities of single-disease, target based management of multiple long term conditions and the overall wellbeing of the individual. Healthcare systems will need to radically change their approaches to meet the challenges and complexity that multimorbidity presents.
